# Nutritional Status, Body Composition and Growth in Paediatric-Onset Ulcerative Colitis: A Systematic Review

**DOI:** 10.3390/nu18010169

**Published:** 2026-01-05

**Authors:** Chen Sarbagili-Shabat, Floor Timmer, Konstantina Morogianni, Ralph de Vries, Tim de Meij, Nikki van der Kruk, Lana Verstoep, Nicolette Wierdsma, Johan Van Limbergen

**Affiliations:** 1 Division of Pediatric Gastroenterology and Nutrition, Department of Pediatrics, Amsterdam University Medical Centers, Emma Children’s Hospital, 1105 AZ Amsterdam, The Netherlands; f.a.timmer@amsterdamumc.nl (F.T.);; 2 Department of Food Science and Human Nutrition, Agricultural University of Athens, 118 55 Athens, Greece; 3 Medical Library, Vrije Universiteit, 1081 HV Amsterdam, The Netherlands; 4 Tytgat Institute for Liver and Intestinal Research, Amsterdam Gastroenterology Endocrinology and Metabolism, 1105 AZ Amsterdam, The Netherlands; 5 Department of Nutrition and Dietetics, Amsterdam University Medical Centers, 1105 AZ Amsterdam, The Netherlands

**Keywords:** inflammatory bowel diseases, ulcerative colitis, body composition, nutritional status, growth, child, adolescent

## Abstract

**Background:** Growth impairment and poor nutritional status are recognized complications of pediatric inflammatory bowel disease (IBD), yet data specific to ulcerative colitis (UC) are limited. This systematic review aims to provide an overview of current knowledge on growth, nutritional status, and body composition in children and adolescents with UC. **Methods:** A systematic literature search was performed up to August 2025. Studies including patients aged 5–22 years with confirmed UC were reviewed. Results related to growth, nutritional status, and body composition were narratively synthesized to summarize findings. **Results:** Fifteen studies with 1575 patients with UC met inclusion criteria, comprising 5 prospective, 5 cross-sectional, and 5 retrospective designs. Although the included studies were conducted in broader IBD cohorts, only UC-specific outcomes were reported. The data were limited by sample size, heterogeneity in patient characteristics, outcome definitions, and assessment methods. The majority of patients had prolonged disease with remission or mild activity. Growth failure prevalence ranged from 7% to 36%, with weight deficits being more common than height deficits. Undernutrition affected up to 25% of patients, with variability across studies. Overweight and obesity were also observed, though most studies showed no significant differences between UC patients and controls. Only five very small studies assessed body composition, reporting inconsistent findings regarding reductions in lean body mass. **Conclusions:** Growth impairment and poor nutritional status can occur in children and adolescents with UC. Larger, standardized, high-quality studies focused specifically on UC are needed to better characterize its impact on growth and nutritional status, including the essential integration of body composition assessment.

## 1. Introduction

Growth impairment and poor nutritional status are prevalent in children with inflammatory bowel diseases (IBD), particularly at diagnosis and during active phases, but can also exist during remission phase [1]. Poor nutritional status reflects an underlying nutritional imbalance and can result in growth impairment, undernutrition, overnutrition, obesity, and altered body composition [2]. Studies have indicated that poor nutritional status reduces quality of life, including a decline in physical performance and social functioning, and is associated with impaired clinical outcomes and an increased need for surgery in patients with IBD [3,4,5]. Although Crohn’s disease (CD) and ulcerative colitis (UC) share some overlapping clinical characteristics, key differences in pathophysiology lead to distinct approaches in disease management [6,7]. These differences are likely to influence nutritional status and growth patterns in distinct ways across conditions. Despite this, many studies group CD and UC together under the umbrella of IBD, which may mask UC-specific variations. Therefore, focusing exclusively on UC allows for a more precise understanding of growth impairment and nutritional features and challenges unique to this population. Two systematic reviews have examined body composition in children with IBD. In one review, reduced lean mass was reported in children with IBD; however, UC represented a minority of participants [8]. A more recent systematic review across chronic inflammatory diseases in children predominantly reported findings in CD, with scarce data for ulcerative colitis [9].

The pathogenesis of UC is thought to result from a dysregulated immune response toward microbial antigens in genetically susceptible individuals exposed to environmental triggers, including dietary factors [10]. UC is characterised by gut microbial dysbiosis, with reduced short-chain fatty acid–producing taxa [11,12] and, in some studies, an increased abundance of potential pathobionts such as hydrogen sulfide–reducing bacteria [12]. Paediatric UC is typically more extensive and severe than adult-onset disease [13]. Assessment of UC in children requires a multidisciplinary approach, incorporating clinical evaluation, endoscopy, imaging, and histological assessment, with disease severity guiding therapeutic decision-making ranging from aminosalicylates and corticosteroids to biologic and immunomodulatory therapies [6,14]. Multiple factors, such as the inflammatory burden, clinical symptoms, changes in dietary habits, physical activity levels, and medical treatments, can influence growth and nutritional status in children and adolescents with UC [15]. Recent nutritional consensus guidelines for IBD emphasize the importance of a thorough nutritional assessment using various tools, including body composition analysis, to accurately evaluate nutritional status. Although linear growth is routinely monitored in clinical practice for patients with IBD, comprehensive evaluation of nutritional status remains challenging due to budget constraints, the need for specialized expertise, and the time required [16]. Relying solely on anthropometric measurements such as body mass index (BMI) may lead to misclassification: overweight or obese patients may appear well-nourished despite being malnourished [4,5]. Furthermore, these measurements alone cannot accurately reflect body composition [17,18,19]. Therefore, the aim of this study is to perform a systematic review to provide an overview of current knowledge on growth, nutritional status, and body composition in children and adolescents with UC.

## 2. Materials and Methods

### 2.1. Systematic Literature Review

The conduct and reporting of this review were in accordance with the Preferred Reporting Items for Systematic Reviews and Meta-Analyses (PRISMA) statement [20]. This study was submitted to the International Prospective Register of Systematic Review—PROSPERO (CRD420251072395) [21].

### 2.2. Search Strategy

To identify the relevant publications, we conducted systematic searches in the bibliographic databases Ovid Medline, Embase.com and Web of Science (Core Collection) covering all records indexed in each database from their start dates up to 12 August 2025. The following terms were used (including synonyms and closely related words) as index terms or free-text words: “Ulcerative Colitis”, “Growth”, “Nutritional Status”, “Malnutrition”, “Body composition”, “Adolescents”, “Children”. The references of the identified articles were searched for relevant publications. Duplicate articles were excluded by a medical information specialist using Endnote X21.0.1 (Clarivate^TM^, London, UK), following the Amsterdam Efficient Deduplication (AED) method, a stepwise approach based on combinations of bibliographic fields to efficiently identify and remove duplicates [22], and the Bramer method, which applies systematic multi-field matching to remove duplicates [23]. The full search strategies for all databases can be found in Supplementary Material (Supplementary Table S1). The main outcomes were related to growth, nutritional status, and body composition, as defined in Table 1.

### 2.3. Selection Process

Two reviewers (CSS and FT) independently screened all potentially relevant titles and abstracts for eligibility. Differences in judgement were resolved through discussion. Studies were included if they met the following criteria: involved children or adolescents aged 5–22 years with an established diagnosis of UC. Although paediatric care typically ends earlier, studies reporting outcomes beyond adolescence were included when disease onset occurred during childhood or adolescence, in order to capture longitudinal growth and nutritional outcomes. Studies were excluded if they included patients with IBD without separate UC data, involved animal models or in vitro research, or were of certain publication types such as editorials, letters, legal cases, interviews, commentaries, or conference abstracts lacking full data. If necessary, the full-text article was checked for the eligibility criteria.

### 2.4. Data Assessment

The full text of the selected articles was obtained for further review. Two reviewers (CSS and FT) independently evaluated the methodological quality of the full-text papers using used the JBI appraisal tools [27]. Any differences between the reviewers were resolved by discussion.

### 2.5. Data Synthesis

Given the anticipated heterogeneity in outcomes (including indicators of overall nutritional status, body composition, and growth), as well as the variability in assessment methods and the relatively small number of studies available for each, we used a qualitative synthesis to present the results. Study characteristics, relevant outcomes, and main results were extracted from the included studies and narratively synthesised to provide broader insights into growth, nutritional status, and body composition in children and adolescents with UC.

## 3. Results

### 3.1. Search Results

**Figure 1:**
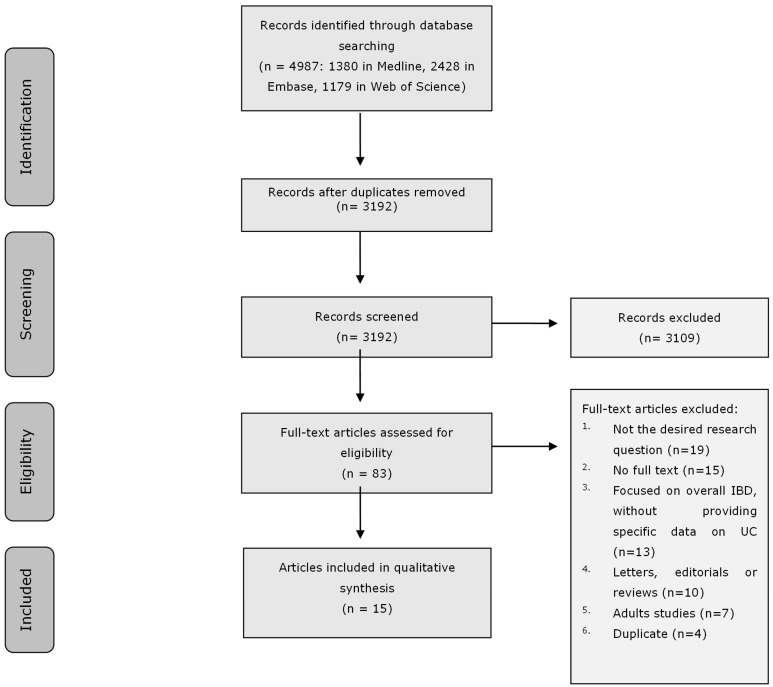
Flow chart of the study selection process for the present systematic review.

The literature search generated a total of 4987 references. After removing duplicates of references that were selected from more than one database, 3192 references remained. The flow chart of the search and selection process is presented in Figure 1. A total of fifteen studies were included: twelve provided data on growth impairment, eight on malnutrition, and five on body composition.

### 3.2. Study and Patient Characteristics

The studies were equally distributed across designs, with 5 prospective, 5 cross-sectional, and 5 retrospective studies (Table 2). The majority of the prospective studies had follow-up periods extending up to one year. The included studies focused on patients with IBD or other gastrointestinal disorders, analyzing UC patients as a subgroup. In total, the included studies reported on 1575 children and adolescents with UC. Sample sizes varied from 12 to 676 participants/patients. The mean or median age across studies varied between 9 and 14 years, with one study reporting a mean age of 21.4 years representing the final measurement [28]. When specified/where reported, the disease duration ranged from less than one year to 6.3 years [28,29,30,31]. Among the six studies that reported disease duration, 63–66% of the UC patients were in remission or had mild active disease [30,31,32,33,34,35]. While most studies did not provide detailed data on medical treatment, three studies reported that patients were treatment-naïve at baseline [33,34,36]. Additionally, in four other studies, aminosalicylates were the most frequently used therapy [29,30,35,37]. Table 2 summarizes only the results reported for patients with UC. Most of the included studies (12/15) did not perform separate analyses for each study outcome or assess statistical significance specifically for patients with UC.

### 3.3. Methodology Assessment of the Outcomes

The included studies in this systematic review employed a variety of standardized methods and criteria to assess growth, nutritional status, and body composition in pediatric UC populations. Growth was predominantly assessed using height and weight converted to z-scores or percentiles according to established reference populations, most commonly the WHO growth standards or national growth references. Definitions of growth impairment varied across studies but typically included height or weight z-scores below specific thresholds (e.g., <−1 or <−1.64) [29,33,34,36,39,42], while several studies used a threshold of −2 standard deviations (SD) to classify growth failure as stunting or short stature [28,30,31,32,33,34,36,37,38,40]. The use of a −2 SD cutoff likely led to an underestimation of growth impairment. Although assessing changes over time is crucial, only a minority of studies incorporated dynamic measures such as height or weight velocity to capture growth patterns over time [28,29]. Nutritional status was primarily evaluated through BMI z-scores to categorize underweight, overweight, or obesity, with cutoffs generally aligned with WHO or national standards [30,31,33,34,37,40].

Body composition was assessed exclusively using indirect, non-invasive methods, mainly bioelectrical impedance analysis (BIA) and anthropometric measures such as mid-upper arm circumference (MUAC), triceps and subscapular skinfold thickness, and handgrip strength [30,31,34,35,41]. Reported parameters included fat mass, fat-free mass, lean body mass, body fat percentage, and phase angle. None of the included studies used dual-energy X-ray absorptiometry (DXA), which is considered the gold-standard method [1], thereby reducing accuracy and sensitivity in detecting subtle changes in muscle and fat mass.

### 3.4. Systematic Evaluation of Growth and Nutritional Status

Across included studies assessing growth, growth failure among pediatric UC patients exhibited variability, with prevalence estimates ranging from 7 to 36%, depending on the definition applied [33,36,39,42]. Zhou et al. reported significantly lower weight percentiles at diagnosis, with growth failure in 30% of boys and 36% of girls, although height did not differ compared with controls. Rates of stunting ranged from 0 to 19% [28,32,36,37,39,40]. The highest prevalence of stunting was reported in a small cohort in Bahrain (19% of 41 UC patients), but no statistical comparison to controls was provided. Weight deficits at diagnosis were commonly reported, with several cohorts identifying a substantial proportion of patients exhibiting weight z-scores below normal thresholds [34,36,38]. In contrast, height impairments were less consistently observed; multiple studies [30,31,35,36,37,38,39] reported no significant differences in height percentiles or height z-scores between patients with UC and healthy controls. Final adult height outcomes were generally comparable to healthy populations, although some evidence suggested that males diagnosed prior to puberty may experience reduced adult stature [28,39].

Malnutrition, including undernutrition, was evaluated with prevalence ranging from 0% to 25%. Two studies, one in Poland and one in the United Kingdom (UK), reported significantly lower BMI z-scores than in the control/reference group despite relatively low rates of growth failure [33]. A study in Saudi Arabia demonstrated a significantly greater prevalence of undernutrition versus the national growth reference, although the difference in growth failure specifically was not significant [40]. A Croatian cohort of newly diagnosed children reported the highest prevalence of undernutrition in UC (25%) [34]. Information on disease severity was lacking in 3 out of 8 studies, while in the other studies, most patients were in remission. In the remaining studies, although most patients were in remission, data comparing active versus inactive disease were not provided, which may partly explain the variability in the results. Overnutrition and obesity were also reported across four cohorts, with three studies showing no significant differences in prevalence compared with controls [31,33,34]. However, overweight prevalence in patients with UC was significantly higher than expected in the Saudi cohort versus reference standards [40].

### 3.5. Systematic Evaluation of Body Composition

Body composition was evaluated in small pediatric or adolescent UC cohorts, with sample sizes ranging from 12 to 40 patients per study [30,31,34,35,41]. Results demonstrated heterogeneity in body composition outcomes, including lean and fat mass parameters among patients with UC. Two studies identified significantly reduced lean mass or phase angle in patients with UC compared with healthy controls [34,35], even in the presence of normal height and weight. Sila et al. specifically reported significantly lower lean body mass. In contrast, Pawłowska-Seredyńska et al. [33] found lean body mass deficiency only in 3% of patients with UC and another study reported no significant difference in lean body mass z-scores versus controls [31].

### 3.6. Quality Assessment

A summary of the methodological quality assessment across included studies is presented in Table 3, with detailed study-level assessments provided in Supplementary Tables S2 and S3. All five cross-sectional studies were found to have a moderate risk of bias. These studies generally provided clear inclusion criteria and used valid and reliable measures for both exposure and condition [31,33,34,35,40]. However, none of the studies identified and addressed cofounding factors, representing the most frequent limitation in this group. In addition, Mouzan et al. and Tsiountsiouria et al. [31,40] failed to describe the subjects in detail.

Among the ten cohort studies, three studies were rated as low risk of bias [36,37,38], six as moderate [28,30,32,39,41,42], and only one as high risk of bias [29]. Most cohort studies adequately measured exposures and outcomes. Again, most studies, except for the studies of Ashton et al. and Jakobsen et al. [37,38] failed to identify cofounding factors. While follow-up duration was often reported as sufficient, incomplete follow-up and lack of strategies to cope with incomplete follow-up contributed to elevated risk of bias in some studies [29,37]. Control groups typically consisted of age and sex matched healthy children from local populations or reference datasets, allowing for comparative analysis. Seven studies compared the UC patients with healthy controls [28,30,31,34,35,39,41], of which five studies used controls matched for age and gender [28,30,35,39,41]. The remaining eight studies used national [29,33,36,37,40,42] or WHO reference populations as comparators [32,38].

### 3.7. Results of Syntheses

All included studies were observational in design and involved relatively small UC subgroups within broader IBD cohorts. Substantial heterogeneity was observed across patient characteristics, outcome definitions, and assessment methods. The included studies indicated higher rates of poor nutritional status and growth impairment at diagnosis [32,34,36,37,39]. However, when disease activity was reported, the majority of included patients were in remission or had mild disease activity, yet still demonstrated a signal of poor nutritional status and growth impairment [30,33,34]. The overall risk of bias was low to moderate, with the most common limitations being inadequate control for confounding factors and incomplete reporting of UC-specific analyses.

## 4. Discussion

In this systematic review, we provide an overview of current knowledge in paediatric-onset UC on growth, nutritional status, and body composition in children and adolescents. Unlike previous related reviews, which primarily evaluated IBD as a whole group, our analysis focused specifically on patients with UC. Our findings showed that most studies assessing growth and nutritional parameters have reported findings in mixed IBD cohorts, often without presenting UC-specific data. Consequently, these results cannot be directly applied to the UC population, limiting disease-specific interpretation. Even among studies that did provide separate UC data, outcome definitions were frequently incomplete, sample sizes were small, and statistical power was rarely addressed. These limitations reduce confidence in the precision of reported associations and highlight the need for larger, UC-focused studies using standardized methods.

Across the included studies, there was substantial heterogeneity in the findings related to the assessment of growth, nutritional status and body composition. These differences can be explained by several factors. First, methodological diversity was evident, including variation in the definitions of growth failure and considerable diversity in the methods used to assess nutritional outcomes. Second, patient and disease characteristics varied across studies. The timing of outcome assessment differed (at diagnosis versus during prolonged disease), and although a higher proportion of patients in most studies were in remission or had mild disease activity, this likely influenced the results, leading to an underestimation of nutritional impairment. Third, differences in control group selection further contributed to heterogeneity, with some studies using matched healthy peers, others relying on general population datasets, and several using WHO or national reference populations.

When considering growth outcomes, most studies reported some degree of impairment among children with UC, with prevalence estimates ranging from 7% to 36%, depending on the criteria applied, with higher rates observed at diagnosis. Weight deficits were more frequently significant than height deficits, suggesting that linear growth impairment is less pronounced in UC. Several longitudinal studies demonstrated that height outcomes often normalized in adolescence or adulthood, particularly with adequate disease control. However, males diagnosed before puberty appeared more vulnerable to reduced final height, suggesting a critical window of risk during early growth. Multiple factors may contribute to impaired growth in UC. Persistent intestinal inflammation can disrupt the growth hormone–IGF-1 pathway, while corticosteroid exposure and reduced nutritional intake during active disease may further impair growth velocity [15]. Growth impairment is generally more severe and prevalent in CD than in UC, largely due to small intestinal involvement, malabsorption, and prolonged inflammation before diagnosis [24]. This difference may, in part, be explained by differences in diagnostic delay and clinical presentation. A large cross-sectional, multicenter Canadian cohort study of 1092 newly diagnosed IBD patients reported a significantly shorter diagnostic delay in those with UC or IBD-unclassified (n = 394) compared with CD [4.7 months, IQR 2.2–10.6 months, vs. 2.9 months, IQR: 1.3–6.5 months, respectively; *p* < 0.001] [43]. This shorter diagnostic delay may partly explain the lower prevalence of linear growth failure observed in UC. The study also found that patients with UC or IBD-unclassified more frequently presented with bloody diarrhea (87%) and experienced a high rate of weight loss (62%), indicating that nutritional deficits can still occur despite relatively preserved height. These findings underscore the importance of comprehensive nutritional monitoring in all patients with IBD. Notably, the study reported a low rate of linear growth failure, with results presented by combining UC and IBD-unclassified cases rather than analysing UC separately. Moreover, the definition of growth failure and the reference standards used were not specified [43].

Our review identified a dual burden of undernutrition and overnutrition in paediatric UC. Undernutrition at diagnosis was common, with prevalence rates of up to 25%, despite the fact that the majority of patients were in remission. Studies from Poland and the UK demonstrated significantly lower BMI z-scores than in controls, despite low stunting rates, suggesting early depletion of body weight relative to height [33,38]. Conversely, overweight and obesity were also reported in several cohorts. While most studies found no significant differences from controls, a study from Saudi Arabia identified a significantly higher prevalence of overweight [40]. This may partly reflect the global rise in obesity, but could also indicate that in certain populations, such as the Saudi cohort, the prevalence of overweight among UC patients is disproportionately higher, potentially influenced by regional dietary or lifestyle factors [44]. Overall, BMI alone may not adequately capture nutritional status in UC, as it can obscure both muscle deficits and excess adiposity, particularly during remission.

Altered body composition represents another important dimension of poor nutritional status in paediatric UC. The studies assessing body composition were limited to small cohorts (n = 12–40) and relied primarily on indirect methods such as BIA and anthropometry, with no studies using the gold-standard DXA [30,31,34,35,41]. Although DXA provides the highest accuracy, its use is limited by low feasibility and the need for specialised equipment. BIA is more practical and accessible for routine care, but its accuracy may be influenced by hydration status and high BMI. Anthropometric and functional measures may complement assessment but cannot reliably diagnose low muscle mass [16]. The wide range of assessment tools and cut-offs used across studies limits comparability and hampers synthesis of the evidence. Despite these limitations, two studies identified significantly reduced lean mass [34,35], even when height and BMI were within normal ranges, suggesting subclinical muscle depletion. In contrast, other studies reported no significant differences in fat mass, fat-free mass, or functional strength compared with healthy controls [31,33]. Longitudinal findings showed improvement in lean tissue indices following disease control [30,41]; however, the magnitude and clinical relevance of these changes remain uncertain. These mixed results and the study limitations emphasize the need for more quality studies to assess body composition in paediatric UC.

**Figure 2:**
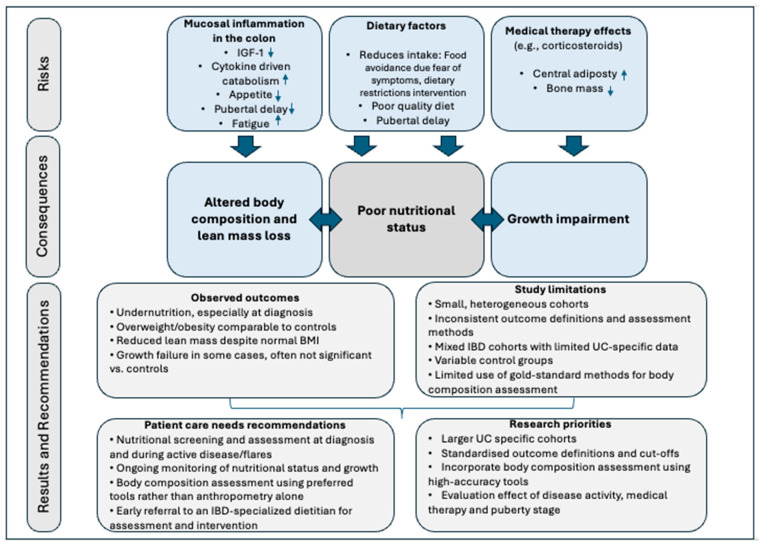
Conceptual framework of poor nutritional status, altered body composition and growth impairment in paediatric ulcerative colitis. Abbreviations: IGF-1, insulin-like growth factor-1; BMI, body mass index; UC, ulcerative colitis; IBD, inflammatory bowel disease.

This systematic review has limitations related to the review process. Due to substantial heterogeneity in study design, outcome definitions, and assessment methods, a quantitative synthesis or meta-analysis was not feasible and results were therefore summarised narratively. In addition, the review is limited the ability to perform stratified analyses by disease severity, disease duration, or treatment exposure. Finally, the lack of access to individual patient data precluded more granular analyses. The multifactorial pathways contributing to poor nutritional status, altered body composition, and growth impairment in paediatric UC are summarised in Figure 2, which serves as a conceptual framework to contextualise both the findings and the limitations of this review.

In conclusion, the limited available evidence suggests that children and adolescents with UC may experience poor nutritional status, manifesting as both undernutrition and overnutrition, as well as growth impairment. Although the available data are heterogeneous and largely observational, and the reported prevalence ranges vary widely and statistical significance was not consistently assessed, the findings across studies suggest a signal of growth impairment and poor nutritional status that may have important implications for patient health. As nutritional status influences both disease outcomes and quality of life, accurate and early detection of these impairments is essential. From a clinical perspective, regular screening for growth impairment and undernutrition at diagnosis is recommended, with continued monitoring of weight, height, and pubertal progression throughout the disease course. Nutritional evaluation should also include body composition and functional strength assessments, with early referral to specialised dietitians for individualised guidance, ensuring adequate macro- and micronutrient intake and promoting overall high-quality dietary patterns. Nutritional management is a key component in the care of paediatric UC, supporting both the prevention of malnutrition and the promotion of optimal growth and development. Based on the findings of this systematic review, future studies should prioritize larger, UC-exclusive cohorts with well-defined control groups, standardized outcome measures, and high-accuracy body composition methodologies. These priorities reflect key methodological gaps consistently identified across the included studies. Moreover, research should consider the influence of disease activity, corticosteroid exposure, biologic therapy, pubertal stage, and ethnicity to better clarify the interplay between disease and nutritional status.

## Figures and Tables

**Table 1:** Definitions and assessment methods of main outcomes.

Definition	Assessment Methods
Growth impairment and growth failure [15,24]	
Growth impairment: A broad term describing any deviation from expected growth patterns, including slowed growth velocity or downward crossing of height percentiles, but not necessarily meeting criteria for short stature. Growth failure: A more severe form of impaired growth, typically defined as a height-for-age z-score significantly below expected (often <−2 SD) and/or a marked reduction in growth velocity compared with predicted or pre-illness growth.	Height-for-age z-score; growth velocity (cm/year); downward crossing of ≥2 major percentiles; comparison with mid-parental target height; serial growth measurements over time
Nutritional status: malnutrition [1,25]	
Malnutrition as deficiency, excess, or imbalance in a person’s intake of energy and/or nutrients	It is typically assessed using anthropometric measures (e.g., BMI z-score), and it is now recommended to complement these with body composition evaluation, particularly lean mass assessment, to better identify compromised nutritional status.
Body composition [26]	
Body composition analysis divides the body into its main tissue compartments: fat mass and fat-free mass, also referred to as lean body mass (LBM). LBM includes all non-adipose tissues, while muscle mass specifically refers to skeletal muscle tissue.	MUAC and MAMC can provide limited insight into muscle mass or visceral adiposity. More advanced techniques, including BIA, DXA, CT, MRI, and ultrasound, provide increasing levels of accuracy for estimating fat mass, fat-free mass, muscle mass, and visceral adiposity.

The definitions shown reflect clinically relevant objectives that are assessed using a combination of objective and quantifiable measures. Abbreviations: SD—Standard Deviation; BMI—Body Mass Index; MUAC—Mid-Upper Arm Circumference; MAMC—Mid-Arm Muscle Circumference; LBM—Lean Body Mass; BIA—Bioelectrical Impedance Analysis; DXA—Dual-energy X-ray Absorptiometry; CT—Computed Tomography; MRI—Magnetic Resonance Imaging.

**Table 2:** Study design characteristics and methodologies for assessing nutritional status in children and adolescents with ulcerative colitis: main results summary of included studies.

First Author, Year (Ref)	Country	Study Design	Study Population (n/Age/Disease Duration/Medication/Severity)	Nutritional Status Relevant Outcome	Method to Assess Nutritional Status Outcome	Control/Reference Group Used	Main Results
Zhou et al., 2024 [36]	Germany	Retrospective, population-based cohort study	N = 130 UC patients out of 421 patients with childhood onset IBD; no specific data on age and severity for the UC group; treatment-naïve at baseline.	Growth impairment	Weight and height z-scores were measured. Z-score < −1 is defined as growth failure and <−2 as short stature. The weight and height scores at diagnosis were converted to percentiles.	General German pediatric population (KiGGS 2003–2006)	UC patients had significantly lower weight at diagnosis (76% < P50, *p* = 0.001) versus the general population. Growth failure was observed in about 30% of the males and 36% of the females, and short stature was observed in 6% of the males and 16% of the females. Statistically significant difference was not reported between UC and general population. There was no significant difference in height percentiles and z-scores between UC patients and the general population.
Pawłowska-Seredyńska et al., 2023 [33]	Poland	Cross-sectional study	N = 29 UC patients out of 70 children with IBD were measured within 3 months from diagnosis; mean age 12.7 ± 2.9 years; most patients (59%) were treatment-naïve, and 65% were in remission or had mild disease activity.	Growth impairment, lean body mass and malnutrition (undernutrition, overnutrition and obesity)	Growth failure was defined as a height z-score < −1.64 and stunting as a height z-score < −2. Underweight was defined as a BMI z-score ≤ −2, overweight as a BMI z-score > 1 and ≤2, and obesity as a BMI z-score > 2. Lean body mass deficiency was defined as MUAC z-score < −2.	Reference population based on a group of healthy children without congenital or developmental disorders	Growth failure and stunting were reported in 6.9% and 3.4% of the UC patients. Underweight, overweight and obesity were observed in 6.9%, 6.9% and 3.5%, respectively, while lean body mass deficiency was present in 3% of the patients. Compared to the reference standard, the BMI z-score was significantly lower (−0.55 ± 0.99).
Isa et al., 2022 [32]	Bahrain	Retrospective, cross-sectional study	N = 41 UC patients out of 88 patients diagnosed with pediatric IBD; mean age at diagnosis was 10.7 ± 3.8 years for the total IBD population; median disease duration was 4.3 years for the total IBD population; Azathioprine, prednisolone and mesalamine therapy were used in most IBD patients (no data for UC); no data on disease severity.	Growth impairment	Growth impairment (stunting) and severe stunting were defined as height z-score < −2 and <−3, respectively.	WHO reference population	Stunting was observed in 19% patients with UC. Severe stunting was observed in 46.7% of the stunted patients in the total IBD population—no UC-specific data
Ashton et al., 2021 [38]	United Kingdom	Retrospective longitudinal study with follow-up up to 5 years	N = 157 UC patients out of 490 IBD patients; mean age 13.0 years at diagnosis; no further data on treatment or disease severity.	Growth impairment and malnutrition (undernutrition)	Stunting was defined as height-SDS < −2 and malnutrition was defined as weight-SDS < −2	WHO reference population	No significant difference in malnutrition rates at diagnosis between the UC group and reference population (2.6% vs. 2.5%). The percentage of stunted patients (1.1%) did not differ from healthy population at diagnosis, 1 year, 2 years and 5 years follow-up.
Assa et al., 2021 [28]	Israel	Retrospective cohort study	N = 676 UC patients out of 2229 patients with childhood onset IBD; mean age 21.4 ± 3.3 of final height/weight measurement, 266 (37%) of all UC patients were diagnosed during their growth potential years (age at diagnosis <16 years for males; <14 years for females); mean disease duration 6.3 years; no further data on medication or disease severity	Growth impairment	Short stature was defined as an adult height z-score < −2. Final adult height and anthropometric z-scores (height, weight, BMI) measurements	Healthy controls N = 1252 with a mean age of 21.3 ± 3.3 years matched by socioeconomic status, sex, and year of birth	There was no difference in short stature rates between UC patients and controls (2.2% vs. 3.9%, *p* = 0.55) No difference in final height was noted between UC patients and controls. UC patients had lower BMI z-score at adulthood compared to controls (males: 0.23 ± 1.25 vs. 0.5 ± 1.38, *p* = 0.005; females: 0.07 ± 1.27 vs. 0.34 ± 1.38, *p* = 0.001). Females had lower weight z-score compared to controls (0.09 ± 1.29 vs. 0.21 ± 1.39, *p* = 0.04)
Rinawi et al., 2020 [39]	Israel	Retrospective longitudinal study providing data at diagnosis and in adulthood (18 years)	N = 125 UC patients out of 291 children with IBD; median age of diagnosis 13.5 years; 34% did not receive steroids during follow-up; no data on disease duration and severity	Growth impairment	Growth impairment defined as height z-score < –1. Growth failure at as height z-score ≤ –2	Healthy controls, N = 125, matched 1:1 by gender and age	No significant difference in prevalence of growth impairment at diagnosis between UC and controls (25.4% vs. 16.9%, *p* = 0.452). Growth failure at diagnosis was also not significantly different (7.2% vs. 3.2%, *p* = 0.358). The mean final adult height was significantly lower among males who were diagnosed prior to final stage of puberty compared with those who were diagnosed post-final stage of puberty
Selbuz et al., 2020 [30]	Turkey	Prospective study with a one-year follow-up	N = 22 UC out of 36 children with IBD; most patients were in remission and were treated with aminosalicylates; no data on disease duration.	Growth impairment, malnutrition (undernutrition) and body composition	Growth failure was defined as height z-score < −2, undernutrition as weight z-score < −2, severe malnutrition as BMI z score < −2. Body composition was assessed using BIA, MUAC and TSF	Healthy control, N = 43, matched by gender and age	Growth failure was observed in 9.1% of UC patients at the end of the study. At baseline, 4.5% of UC patients were undernourished, and 13.6% of these patients were severely malnourished. By the end of the study, 4.5% remained undernourished, and 9.1% were severely malnourished. Compared to matched controls, changes in anthropometrics and body composition parameters during 1-year follow-up did not differ significantly, except for an increase in the triceps skinfold thickness z score. Both FM and FFM significantly improved significantly over time
El Mouzan et al., 2020 [40]	Saudi Arabia	Cross-sectional study	N= 119 UC patients out of 374 children and adolescents with pediatric IBD; mean age at diagnosis was 9.1 SD years (no data for UC patients); extensive colitis was the commonest extent at diagnosis (58%); no data on medication or disease duration and severity	Growth impairment and malnutrition (undernutrition and overnutrition)	Short stature was defined as Height z-score < −2 Thinness (underweight) was defined as BMI z-score < −2 Overweight (including obesity) was defined as BMI z-score > 1	Saudi National Growth Reference and WHO reference population	Based on the national growth reference, the prevalence of thinness, overweight, and short stature in the UC group was 8% (*p* = 0.005), 20% (*p* = 0.004), and 12% (*p* = 0.91), respectively. Using the WHO reference, the prevalence of thinness, overweight, and short stature in the UC group was significantly higher at 24%, 20%, and 21%, respectively.
Sila et al., 2019 [34]	Croatia	Cross-sectional study	N = 40 UC patients out of 89 newly diagnosed children with IBD; mean age 14.0 ± 3.7 years; most patients (59%) had not received any medication, and 65% were in remission or had mild disease activity.	Malnutrition (undernutrition and overnutrition) and body composition	Mild malnutrition was defined as a BMI z-score between −1 and −2, while moderate and severe malnutrition were defined as a BMI z-score ≤ −2. Overweight was defined as BMI z-score ≥ 1, and obesity as ≥2 Body composition was assessed using BIA, MUAC, TSF, SSF and HGS.	Healthy control N = 159 with the mean age 14.7 years	Compared to healthy controls, significantly lower lean body mass for age z-scores were found. TSF, SSF, MUAC, and body fat percentages did not differ significantly from healthy controls. At diagnosis, 25% of UC patients were undernourished, including 10% moderately to severely malnourished, compared to 1.9% in controls. Overweight was seen in 17.5% of UC patients vs. 21.4% in controls, with no UC patients classified as obese, compared to 8.2% of controls.
Więch et al., 2017 [41]	Poland	Prospective study with a one-year follow-up	N = 16 newly diagnosed UC patients out of 59 IBD patients of which n = 9 were assessed again 1 year after their UC diagnosis; mean age 13.5 years; medication not specified; most patients had mild to moderate disease.	Body composition	Body composition, including FM, FFM, BCM, MM, TBW, was assessed using BIA.	Healthy controls N = 16 matched by age and sex	Compared to healthy controls, UC patients did not differ significantly in FM, FFM, MM, BCM, and TBM. After one year, all the selected components of body composition were increased significantly, except for the FM.
Tsiountsioura et al., 2014 [31]	United Kingdom	Cross-sectional study	N = 27 pediatric UC patients out of 168 patients from outpatient gastroenterology clinics; median age 12.2 years; disease duration and medication not detailed; 63% had inactive disease.	Growth impairment, malnutrition (undernutrition and overnutrition) and body composition	Short stature was defined as height z-score < −2. Participant with a BMI z-score < −2 were classified as thin and with BMI z-score > 2 as obese. Body composition was assessed using BIA, TSF, and HGS.	Healthy controls N = 62, median age 9.8 years	No significant differences were found between UC patients and controls in the prevalence of short stature (3.7% vs. 4.9%), BMI z-scores (0.8 vs. 0.3), prevalence of thinness (0 vs.4.9) or prevalence of obesity (19% vs. 15%, respectively). Compared to controls, there was no significant difference in lean mass z-scores, fat mass z-scores, TSF z-scores and grip strength for height.
Werkstetter et al., 2012 [35]	Germany	Cross-sectional study	N = 12 UC patients out of 39 patients with IBD; median age at diagnosis 11.7 years; median disease duration 3.1 years; All the patients have used glucocorticoids at some point, most patients were treated with either aminosalicylates or azathiopirine; 66% were in remission.	Body composition and malnutrition	Height, weight, and BMI z-scores were measured. Body composition was assessed using BIA to measure the phase angle α as indicator of lean body mass. In addition, grip strength was measured.	Healthy controls N = 39 matched by age and sex	UC patients had significantly reduced phase angle α compared to controls. No differences between patients and healthy controls in height, weight, BMI, and hand grip strength.
Jakobsen et al., 2011 [37]	Denmark	Prospective cohort study with a median follow-up of 511 days (IQR 191–1053)	N = 62 UC out of 130 patients (<15 years) with IBD; Mean age at diagnosis was 12.4 years; medication used (in proportions of patients): 5-ASA (87.1%), corticosteroids (1.6%), Immunomodulators (33.9%), biological treatment (17.7%); no data on disease duration or severity.	Growth impairment and malnutrition (undernutrition)	Growth retardation was defined as height z-score < −2. Malnutrition was defined as BMI z-score < −2.	Healthy Danish pediatric reference population	No UC patients had weight or height z-score < −2, but 11.5% were considered malnourished at diagnosis (no follow-up results on growth and nutritional status available).
Lee et al., 2010 [42]	United States of America	Prospective cohort study with a mean follow-up of 2.3 SD years from the time of diagnosis	N = 84 UC patients out of 295 IBD patients; mean age at enrollment was 13.9 for the total IBD population (no data for UC); Corticosteroids, Immunosuppressive therapy, Biologic therapy, Nutrition therapy were used in most patients (no data for UC); 73.8% of UC patients had extensive involvement at enrollment; no further data on disease duration or severity	Growth impairment	Growth impairment was defined as height for age z-score < −1.64 in more than one measurement since diagnosis.	National Center for Health Statistics general population reference	Growth impairment was observed in 12% of patients with UC. Other outcomes were outside the scope of this review.
Motil et al., 1993 [29]	United States of America	Prospective study with a short-term follow-up of one year and a long-term follow-up of up to three years.	N = 35 UC out of 70 children with IBD; mean age 12.0 years; Seventy-four percent received sulfasalazine and 34% corticosteroids as treatment. Disease duration was less than one year in 49% of the patients. Most patients were in remission, experiencing no abdominal pain and having normal bowel movements.	Growth impairment	Growth failure was defined as height and weight z-scores < −1.64, height less than 95% of the expected value at the 50th percentile for age, or weight less than 90% of the expected value at the 50th percentile for age or height. Additionally, growth failure included height velocity less than 4 cm per year for males under 15 years and females under 13 years, as well as weight velocity less than 1.0 kg per year for males under 15 years and females under 13 years.	National growth reference	Growth failure was observed in 23% of children with UC based on height-for-age measurements, in 9% based on height Z scores, in 31% based on weight-for-age, in 14% based on weight-for-height, and in 6% based on weight Z scores. Growth failure assessed by height and weight velocity was identified in 16% and 6% of patients, respectively. No significant differences in height or weight Z scores were found between baseline and long-term follow-up.

Abbreviations: UC—Ulcerative colitis; IBD—Inflammatory bowel disease; P—percentile; BMI—Body Mass Index; WHO—World Health Organization; SDS—Standard Deviation Score; BIA—Bioelectrical Impedance Analysis; FM—Fat Mass; FFM—Fat-Free Mass; HGS—Handgrip Strength; MUAC—Mid-Upper Arm Circumference; TSF—Triceps Skinfold Thickness; SSF—Subscapular Skinfold Thickness; BCM—Body Cell Mass; MM—Muscle Mass; TBW—Total Body Water; 5-ASA—5-Aminosalicylic acid.

**Table 3:** Summary of quality assessment across included studies.

Domain	Cross-Sectional Studies (n = 5)	Cohort Studies (n = 10)	Key Limitations
Population & setting	Mostly adequate	Mostly adequate	Limited UC-specific detail
UC definition & outcome measurement	Adequate	Adequate	Outcome measurement: the wide range of tools and cut-offs used across studies limits comparability and hampers synthesis of the evidence
Confounding identified and addressed	Rarely	Inconsistently	Limited identification of potential confounders, including disease activity, treatment exposure, pubertal stage, age, and other patient and clinical characteristics
Follow-up and completeness	Not applicable	Variable	Incomplete reporting of follow-up duration and loss to follow-up
Statistical analysis	Generally appropriate	Generally appropriate	Limited UC-specific analyses in mixed IBD cohorts

Detailed JBI critical appraisal results for individual studies are provided in Supplementary Tables S2 and S3. Abbreviations: UC, ulcerative colitis; IBD, inflammatory bowel disease.

## Data Availability

The original contributions presented in this study are included in the article and Supplementary Materials. Further inquiries can be directed to the corresponding author.

## Supplementary Materials

The following supporting information can be downloaded at: https://www.mdpi.com/article/10.3390/nu18010169/s1, Supplementary Table S1. Search strategies for all databases, Supplementary Table S2. Quality assessment for cross-sectional studies, Supplementary Table S3. Quality assessment for cohort studies.

## References

[1] Hill R.J. (2014). Update on nutritional status, body composition and growth in paediatric inflammatory bowel disease. World J. Gastroenterol..

[2] Bischoff S.C., Bager P., Escher J., Forbes A., Hebuterne X., Hvas C.L., Joly F., Klek S., Krznaric Z., Ockenga J. (2023). ESPEN guideline on Clinical Nutrition in inflammatory bowel disease. Clin. Nutr..

[3] Liu S., Ding X., Maggiore G., Pietrobattista A., Satapathy S.K., Tian Z., Jing X. (2022). Sarcopenia is associated with poor clinical outcomes in patients with inflammatory bowel disease: A prospective cohort study. Ann. Transl. Med..

[4] Norman K., Kirchner H., Lochs H., Pirlich M. (2006). Malnutrition affects quality of life in gastroenterology patients. World J. Gastroenterol..

[5] Prince A.C., Moosa A., Lomer M.C., Reidlinger D.P., Whelan K. (2015). Variable access to quality nutrition information regarding inflammatory bowel disease: A survey of patients and health professionals and objective examination of written information. Health Expect..

[6] Assa A., Aloi M., Van Biervliet S., Bronsky J., di Carpi J.M., Gasparetto M., Gianolio L., Gordon H., Hojsak I., Hudson A.S. (2025). Management of paediatric ulcerative colitis, part 2: Acute severe colitis-An updated evidence-based consensus guideline from the European Society of Paediatric Gastroenterology, Hepatology and Nutrition and the European Crohn’s and Colitis Organization. J. Pediatr. Gastroenterol. Nutr..

[7] van Rheenen P.F., Aloi M., Assa A., Bronsky J., Escher J.C., Fagerberg U.L., Gasparetto M., Gerasimidis K., Griffiths A., Henderson P. (2021). The Medical Management of Paediatric Crohn’s Disease: An ECCO-ESPGHAN Guideline Update. J. Crohns. Colitis.

[8] Thangarajah D., Hyde M.J., Konteti V.K., Santhakumaran S., Frost G., Fell J.M. (2015). Systematic review: Body composition in children with inflammatory bowel disease. Aliment. Pharmacol. Ther..

[9] Houttu N., Kalliomaki M., Gronlund M.M., Niinikoski H., Nermes M., Laitinen K. (2020). Body composition in children with chronic inflammatory diseases: A systematic review. Clin. Nutr..

[10] Swirkosz G., Szczygiel A., Logon K., Wrzesniewska M., Gomulka K. (2023). The Role of the Microbiome in the Pathogenesis and Treatment of Ulcerative Colitis—A Literature Review. Biomedicines.

[11] James S.L., Christophersen C.T., Bird A.R., Conlon M.A., Rosella O., Gibson P.R., Muir J.G. (2015). Abnormal fibre usage in UC in remission. Gut.

[12] Yao C.K., Sarbagili-Shabat C. (2023). Gaseous metabolites as therapeutic targets in ulcerative colitis. World J. Gastroenterol..

[13] Van Limbergen J., Russell R.K., Drummond H.E., Aldhous M.C., Round N.K., Nimmo E.R., Smith L., Gillett P.M., McGrogan P., Weaver L.T. (2008). Definition of phenotypic characteristics of childhood-onset inflammatory bowel disease. Gastroenterology.

[14] Wine E., Aloi M., Van Biervliet S., Bronsky J., di Carpi J.M., Gasparetto M., Gianolio L., Gordon H., Hojsak I., Hudson A.S. (2025). Management of paediatric ulcerative colitis, part 1: Ambulatory care-An updated evidence-based consensus guideline from the European Society of Paediatric Gastroenterology, Hepatology and Nutrition and the European Crohn’s and Colitis Organisation. J. Pediatr. Gastroenterol. Nutr..

[15] Wong K., Isaac D.M., Wine E. (2021). Growth Delay in Inflammatory Bowel Diseases: Significance, Causes, and Management. Dig. Dis. Sci..

[16] Svolos V., Gordon H., Lomer M.C.E., Aloi M., Bancil A., Day A.S., Day A.S., Fitzpatrick J.A., Gerasimidis K., Gkikas K. (2025). European Crohn’s and Colitis Organisation consensus on dietary management of inflammatory bowel disease. J. Crohns. Colitis.

[17] Adams D.W., Gurwara S., Silver H.J., Horst S.N., Beaulieu D.B., Schwartz D.A., Seidner D.L. (2017). Sarcopenia Is Common in Overweight Patients with Inflammatory Bowel Disease and May Predict Need for Surgery. Inflamm. Bowel Dis..

[18] Abd-El-Aziz M.A., Hubner M., Demartines N., Larson D.W., Grass F. (2022). Simple Clinical Screening Underestimates Malnutrition in Surgical Patients with Inflammatory Bowel Disease-An ACS NSQIP Analysis. Nutrients.

[19] Bryant R.V., Ooi S., Schultz C.G., Goess C., Grafton R., Hughes J., Lim A., Bartholomeusz F.D., Andrews J.M. (2015). Low muscle mass and sarcopenia: Common and predictive of osteopenia in inflammatory bowel disease. Aliment. Pharmacol. Ther..

[20] Page M.J., McKenzie J.E., Bossuyt P.M., Boutron I., Hoffmann T.C., Mulrow C.D., Shamseer L., Tetzlaff J.M., Akl E.A., Brennan S.E. (2021). The PRISMA 2020 statement: An updated guideline for reporting systematic reviews. BMJ.

[21] PROSPERO: International Prospective Register of Systematic Reviews. University of York. https://www.crd.york.ac.uk/prospero/.

[22] Otten R., de Vries R., Schoonmade L. (2019). Amsterdam Efficient Deduplication (AED) method—Manual. Zenodo.org. https://zenodo.org/records/4544315.

[23] Bramer W.M., Giustini D., de Jonge G.B., Holland L., Bekhuis T. (2016). De-duplication of database search results for systematic reviews in EndNote. J. Med. Libr. Assoc..

[24] Ishige T. (2019). Growth failure in pediatric onset inflammatory bowel disease: Mechanisms, epidemiology, and management. Transl. Pediatr..

[25] Cederholm T., Jensen G.L., Correia M., Gonzalez M.C., Fukushima R., Higashiguchi T., Baptista G., Barazzoni R., Blaauw R., Coats A. (2019). GLIM criteria for the diagnosis of malnutrition—A consensus report from the global clinical nutrition community. J. Cachexia Sarcopenia Muscle.

[26] Ding N.S., Tassone D., Al Bakir I., Wu K., Thompson A.J., Connell W.R., Malietzis G., Lung P., Singh S., Choi C.R. (2022). Systematic Review: The Impact and Importance of Body Composition in Inflammatory Bowel Disease. J. Crohns. Colitis.

[27] Moola S., Munn Z., Tufanaru C., Aromataris E., Sears K., Sfetcu R., Currie M., Lisy K., Qureshi R., Mattis P. (2020). Systematic reviews of etiology and risk. JBI Manual for Evidence Synthesis.

[28] Assa A., Assayag N., Balicer R.D., Gabay H., Greenfeld S., Kariv R., Ledderman N., Matz E., Dotan I., Ledder O. (2021). Pediatric-onset Inflammatory Bowel Disease Has Only a Modest Effect on Final Growth: A Report From the epi-IIRN. J. Pediatr. Gastroenterol. Nutr..

[29] Motil K.J., Grand R.J., Davis-Kraft L., Ferlic L.L., Smith E.O. (1993). Growth failure in children with inflammatory bowel disease: A prospective study. Gastroenterology.

[30] Selbuz S., Kansu A., Berberoglu M., Siklar Z., Kuloglu Z. (2020). Nutritional status and body composition in children with inflammatory bowel disease: A prospective, controlled, and longitudinal study. Eur. J. Clin. Nutr..

[31] Tsiountsioura M., Wong J.E., Upton J., McIntyre K., Dimakou D., Buchanan E., Cardigan T., Flynn D., Bishop J., Russell R.K. (2014). Detailed assessment of nutritional status and eating patterns in children with gastrointestinal diseases attending an outpatients clinic and contemporary healthy controls. Eur. J. Clin. Nutr..

[32] Isa H.M., Mohamed M.S., Alahmed F.A., Mohamed A.M. (2022). Linear Growth Impairment in Patients with Pediatric Inflammatory Bowel Disease. Cureus.

[33] Pawłowska-Seredyńska K., Akutko K., Umlawska W., Smieszniak B., Seredynski R., Stawarski A., Pytrus T., Iwanczak B. (2023). Nutritional status of pediatric patients with inflammatory bowel diseases is related to disease duration and clinical picture at diagnosis. Sci. Rep..

[34] Sila S., Trivic I., Pavic A.M., Niseteo T., Kolacek S., Hojsak I. (2019). Nutritional status and food intake in pediatric patients with inflammatory bowel disease at diagnosis significantly differs from healthy controls. Eur. J. Pediatr..

[35] Werkstetter K.J., Ullrich J., Schatz S.B., Prell C., Koletzko B., Koletzko S. (2012). Lean body mass, physical activity and quality of life in paediatric patients with inflammatory bowel disease and in healthy controls. J. Crohns. Colitis.

[36] Zhou X., Kern I., Rothe U., Schoffer O., Weidner J., Richter T., Laass M.W., Kugler J., Manuwald U. (2024). Growth development of children and adolescents with inflammatory bowel disease in the period 2000–2014 based on data of the Saxon pediatric IBD registry: A population-based study. BMC Gastroenterol..

[37] Jakobsen C., Paerregaard A., Munkholm P., Faerk J., Lange A., Andersen J., Jakobsen M., Kramer I., Czernia-Mazurkiewicz J., Wewer V. (2011). Pediatric inflammatory bowel disease: Increasing incidence, decreasing surgery rate, and compromised nutritional status: A prospective population-based cohort study 2007–2009. Inflamm. Bowel Dis..

[38] Ashton J.J., Green Z., Young A., Borca F., Coelho T., Batra A., Afzal N.A., Ennis S., Johnson M.J., Beattie R.M. (2021). Growth failure is rare in a contemporary cohort of paediatric inflammatory bowel disease patients. Acta Paediatr..

[39] Rinawi F., Assa A., Almagor T., Ziv-Baran T., Shamir R. (2020). Prevalence and Predictors of Growth Impairment and Short Stature in Pediatric-Onset Inflammatory Bowel Disease. Digestion.

[40] El Mouzan M., Alahmadi N., ALSaleeem K.A., Assiri A., AlSaleem B., Al Sarkhy A. (2020). Prevalence of nutritional disorders in Saudi children with inflammatory bowel disease based on the national growth reference. Arab. J. Gastroenterol..

[41] Więch P., Binkowska-Bury M., Korczowski B. (2017). Body composition as an indicator of the nutritional status in children with newly diagnosed ulcerative colitis and Crohn’s disease—A prospective study. Prz. Gastroenterol..

[42] Lee J.J., Escher J.C., Shuman M.J., Forbes P.W., Delemarre L.C., Harr B.W., Kruijer M., Moret M., Allende-Richter S., Grand R.J. (2010). Final adult height of children with inflammatory bowel disease is predicted by parental height and patient minimum height Z-score. Inflamm. Bowel Dis..

[43] Ricciuto A., Mack D.R., Huynh H.Q., Jacobson K., Otley A.R., deBruyn J., El-Matary W., Deslandres C., Sherlock M.E., Critch J.N. (2021). Diagnostic Delay Is Associated With Complicated Disease and Growth Impairment in Paediatric Crohn’s Disease. J. Crohns. Colitis.

[44] Alhusseini N., Alsinan N., Almutahhar S., Khader M., Tamimi R., Elsarrag M.I., Warar R., Alnasser S., Ramadan M., Omair A. (2023). Dietary trends and obesity in Saudi Arabia. Front. Public Health.

