# Supplementary material for: Nutritional Status, Body Composition and Growth in Paediatric-Onset Ulcerative Colitis: A Systematic Review
Source: Nutrients. 2026 Jan 5;18(1):169. doi: 10.3390/nu18010169 (PMC12787959; doi:10.3390/nu18010169)
Supplement: Supplementary file 1 [file nutrients-18-00169-s001.zip › nutrients-4034773-supplementary.pdf]

Supplementary Table S1. Search strategies for all databases

Ovid Medline Search Results – 12 Aug 2025 (https://tools.ovid.com/ovidtools/medline/)

Search	Query	Results
#4	1 and 2 and 3	1,380
#3	"Adolescent"/ or exp "Child"/ or ("adolescen*" or "child*" or "schoolchild*" or "girl*" or "boy*" or "teen" or "teens" or "teenager*" or "youth*" or "pediatr*" or "paediatr*" or "puber*" or "juvenil*").ab,ti,kf.	4,445,626
#2	"Nutritional Status"/ or exp "Body Composition"/ or "Growth and Development"/ or exp "Growth"/ or exp "Human Development"/ or exp "Overweight"/ or exp "Body Weights and Measures"/ or "Sarcopenia"/ or exp "Malnutrition"/ or "Child Nutrition Disorders"/ or ((("nutri*" or "diet*") adj3 ("stat*" or "intake*" or "pattern*" or "deficien*" or "insuf*")) or "eutrophia" or "body composition" or "body weight*" or "body mass" or "muscle mass" or "BMI" or "growth" or "overweight" or "obes*" or "sarcopen*" or "malnutrition" or "undernutrition" or "malnourish*").ab,ti,kf.	3,763,990
#1	"Colitis, Ulcerative"/ or ("ulcerative colitis" or "idiopathic proctocolitis" or "colitis gravis" or "colitis ulcer*" or "mucosal colitis" or "ulcerative colorectitis" or "ulcerative proctocolitis" or "ulcerative procto-colitis" or "ulcerous colitis" or "chronic ulceration" or (("pediatric" or "paediatric" or "child*" or "adolescen*") adj3 "inflammatory bowel")).ab,ti,kf.	69,192

Embase.com Search Results – 12 Aug 2025 (https://www.embase.com)

Search	Query	Results
#5	#4 NOT ('conference abstract'/it OR 'conference review'/it) NOT 'clinical trial':dtype	2,428
#4	#1 AND #2 AND #3	3,778

Search	Query	Results
#3	'juvenile'/de OR 'adolescent'/exp OR 'child'/exp OR ("adolescen*" OR "child*" OR "schoolchild*" OR "girl*" OR "boy*" OR "teen" OR "teens" OR "teenager*" OR "youth*" OR "pediatr*" OR "paediatr*" OR "puber*" OR "juvenil*"):ab,ti,kw	5,719,683
#2	'nutritional status'/exp OR 'body composition'/exp OR 'growth, development and aging'/de OR 'growth'/exp OR 'human development'/de OR 'adolescent development'/exp OR 'child development'/exp OR 'obesity'/exp OR 'sarcopenia'/exp OR 'malnutrition'/exp OR 'nutritional deficiency'/exp OR ((("nutri*" OR "diet*") NEAR/3 ("stat*" OR "intake*" OR "pattern*" OR "deficien*" OR "insuf*")) OR "eutrophia" OR "body composition" OR "body weight*" OR "body mass" OR "muscle mass" OR "BMI" OR "growth" OR "overweight" OR "obes*" OR "sarcopen*" OR "malnutrition" OR "undernutrition" OR "malnourish*"):ab,ti,kw	5,623,816
#1	'ulcerative colitis'/exp OR ("ulcerative colitis" OR "idiopathic proctocolitis" OR "colitis gravis" OR "colitis ulcer*" OR "mucosal colitis" OR "ulcerative colorectitis" OR "ulcerative proctocolitis" OR "ulcerative procto-colitis" OR "ulcerous colitis" OR "chronic ulceration" OR (("pediatric" OR "paediatric" OR "child*" OR "adolescen*") NEAR/3 "inflammatory bowel")):ab,ti,kw	130,145

Web of Science (Core Collection) Search Results – 12 Aug 2025

(https://www.webofscience.com/wos/)

Search	Query	Results
#4	#1 AND #2 AND #3	1,179
#3	TS=("adolescen*" OR "child*" OR "schoolchild*" OR "girl*" OR "boy*" OR "teen" OR "teens" OR "teenager*" OR "youth*" OR "pediatr*" OR "paediatr*" OR "puber*" OR "juvenil*")	3,712,425
#2	TS=((("nutri*" OR "diet*") NEAR/3 ("stat*" OR "intake*" OR "pattern*" OR "deficien*" OR "insuf*")) OR "eutrophia" OR "body composition" OR "body weight*" OR "body mass" OR "muscle mass" OR "BMI" OR	5,781,463

Search	Query	Results
	**"growth" OR "overweight" OR "obes*" OR "sarcopen*" OR "malnutrition" OR "undernutrition" OR "malnourish*")**	
#1	**TS=("ulcerative colitis" OR "idiopathic proctocolitis" OR "colitis gravis" OR "colitis ulcer*" OR "mucosal colitis" OR "ulcerative colorectitis" OR "ulcerative proctocolitis" OR "ulcerative procto-colitis" OR "ulcerous colitis" OR "chronic ulceration" OR (("pediatric" OR "paediatric" OR "child*" OR "adolescen*") NEAR/3 "inflammatory bowel"))**	89,052

Supplementary Table S2. Quality assessment for cross-sectional studies

Study	Q1	Q2	Q3	Q4	Q5	Q6	Q7	Q8	Overall
Sila *et al*, 2019 [34]	+	-	+	+	-	NA	+	+	Moderate
Pawłowska-Seredyńska *et al*, 2023 [33]	+	+	+	+	-	NA	-	+	Moderate
Mouzan *et al*, 2020 [40]	+	-	+	+	-	NA	-	+	Moderate
Tsiountsioura et al., 2014 [31]	+	-	+	+	-	NA	+	+	Moderate
Werkstetter et al., 2012 [35]	+	+	+	+	-	NA	+	+	Moderate

+ = yes; - = no; ? = unclear; NA = not applicable

Overall risk of bias: high = few criteria are met, or major methodological flaws exist; moderate = several criteria are met; but some key elements are missing, low = most or all criteria are met and no critical items are missing.

Questions:
1.Were the criteria for inclusion in the sample clearly defined? 2.Were the study subjects and the setting described in detail? 3.Was the exposure measured in a valid and reliable way? 4.Were objective, standard criteria used for measurement of the condition? 5.Were confounding factors

identified? 6.Were strategies to deal with confounding factors stated? 7.Were the outcomes measured in a valid and reliable way? 8.Was appropriate statistical analysis used?

Supplementary Table S3. Quality assessment for cohort studies

Study	Q1	Q2	Q3	Q4	Q5	Q6	Q7	Q8	Q9	Q10	Q11	Overall
Rinawi *et al*, 2020 [39]	+	+	+	-	NA	-	+	+	+	NA	+	Moderate
Selbuz *et al*, 2020 [30]	+	+	+	-	NA	-	+	-	+	NA	+	Moderate
Motil *et al*, 1993 [29]	+	NA	?	-	NA	-	+	+	-	-	+	High
Assa *et al*, 2021 [28]	+	+	+	-	NA	NA	+	+	+	NA	+	Moderate
Jakobsen *et al*, 2011 [37]	+	+	+	+	+	-	+	+	-	-	+	Low
Isa *et al*, 2022 [32]	-	NA	+	-	NA	-	+	+	+	NA	+	Moderate
Lee *et al,* 2010 [42]	+	NA	+	-	NA	-	+	+	+	NA	+	Moderate
Więch et al, 2017 [41]	+	+	+	-	-	-	+	+	+	-	+	Moderate
Zhou et al, 2024 [36]	+	+	+	-	-	-	+	+	+	+	+	Low
Ashton et al, 2021 [38]	-	NA	+	+	+	-	+	+	+	+	+	Low

+ = yes; - = no; ? = unclear; NA = not applicable

Overall risk of bias: high = few criteria are met or major methodological flaws exist, moderate = several criteria are met, but some key elements are missing, low = most or all criteria are met and no critical items are missing.

Questions:

1.Were the two groups similar and recruited from the same population? 2.Were the exposures measured similarly to assign people to both exposed and unexposed groups? 3.Was the exposure measured in a valid and reliable way? 4.Were confounding factors identified? 5.Were strategies to deal with confounding factors stated? 6.Were the groups/participants free of the outcome at the start of the study (or at the moment of exposure)? 7.Were the outcomes measured in a valid and reliable way? 8.Was the follow up time reported and sufficient to be long enough for outcomes to occur? 9.Was follow up complete, and if not, were the reasons to loss to follow up described and explored? 10.Were strategies to address incomplete follow up utilized?11. Was appropriate statistical analysis used?
